# Supplementary material for: Crowd-sourced observations of a polyphagous moth reveal evidence of allochronic speciation varying along a latitudinal gradient
Source: PLoS One. 2023 Jul 13;18(7):e0288415. doi: 10.1371/journal.pone.0288415 (PMC10343147; doi:10.1371/journal.pone.0288415)
Supplement: S1 Table — Among the coefficients of linear discriminants, the mean green value, mean red value, and red standard deviation have the highest absolute values (-2.60, 1.75, and -0.97 respectively). (DOCX) [file pone.0288415.s001.docx]

| Variable | Value |
| --- | --- |
| GreenMean | -2.60732 |
| RedMean | 1.750011 |
| RedSD | -0.96515 |
| BlueMax | 0.796165 |
| RedMin | 0.7282 |
| GreenMax | -0.71824 |
| BlueSD | 0.656008 |
| BlueSkew | -0.65438 |
| RedSkew | 0.638823 |
| GreenKurt | 0.512545 |
| RedKurt | -0.3341 |
| GreenMin | -0.3192 |
| RedMax | 0.300597 |
| GreenSkew | 0.163374 |
| BlueMin | -0.112 |
| BlueKurt | -0.1039 |
| GreenSD | 0.089111 |
| BlueMean | 0.077523 |
