# Supplementary figures and images for: Crowd-sourced observations of a polyphagous moth reveal evidence of allochronic speciation varying along a latitudinal gradient
Source: PLoS One. 2023 Jul 13;18(7):e0288415. doi: 10.1371/journal.pone.0288415 (PMC10343147; doi:10.1371/journal.pone.0288415)

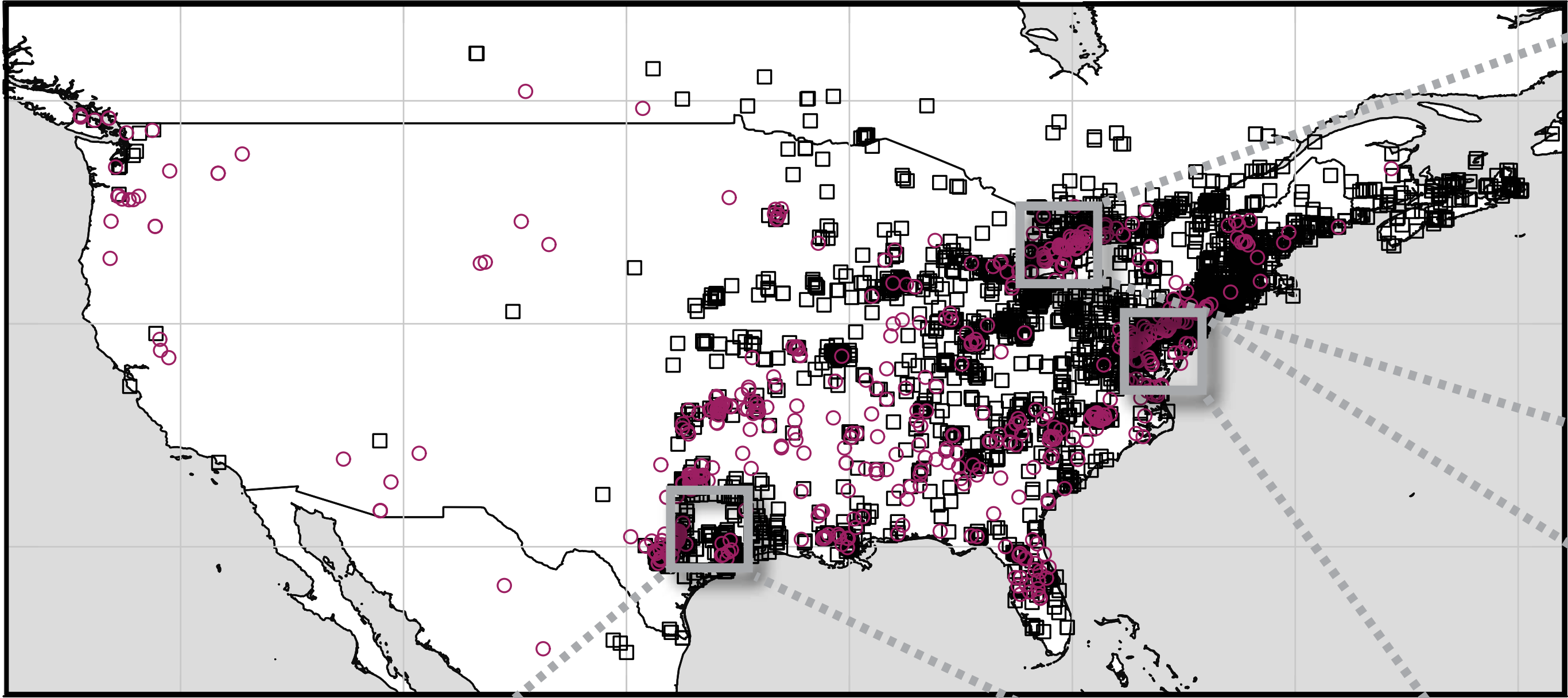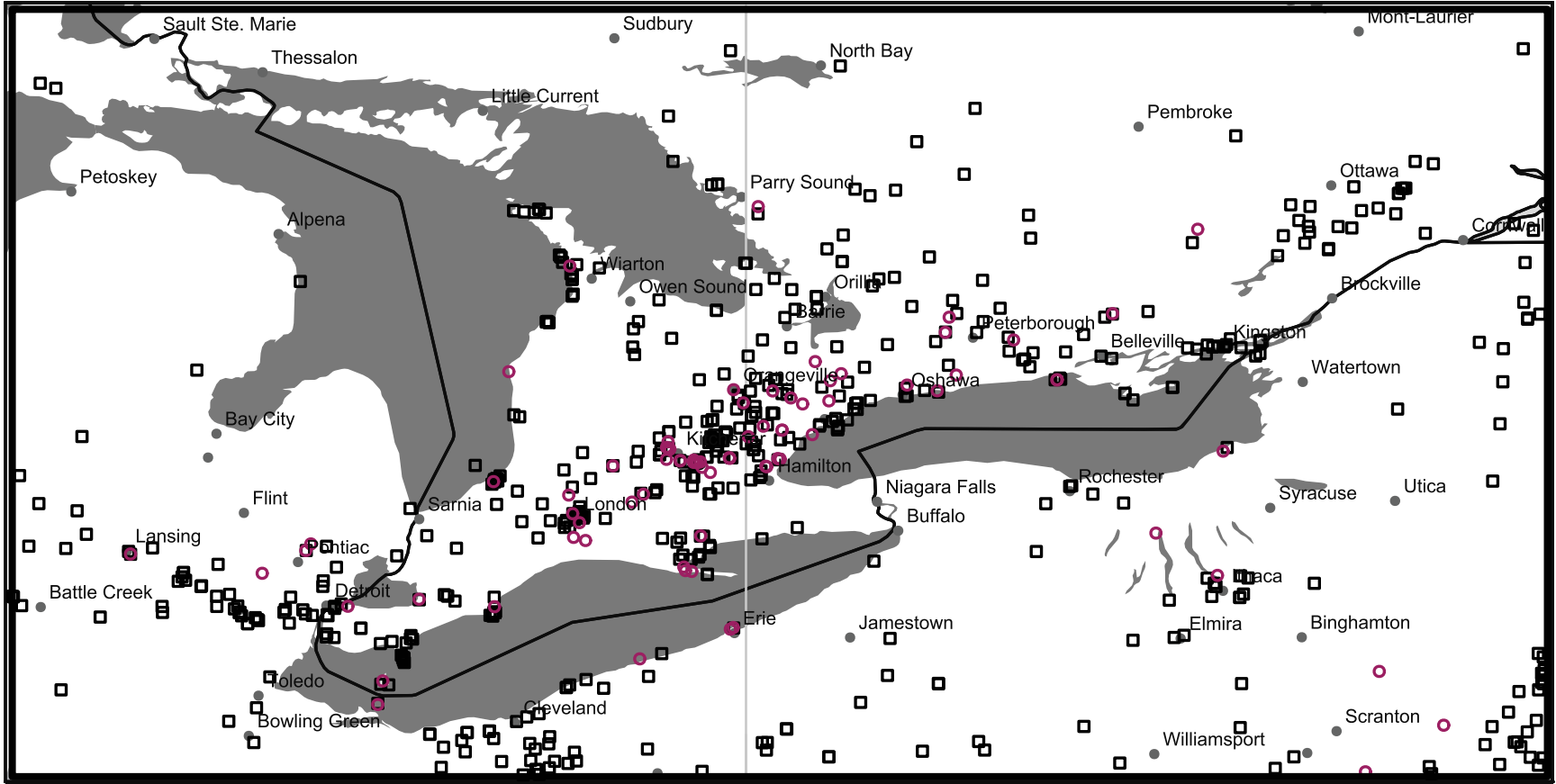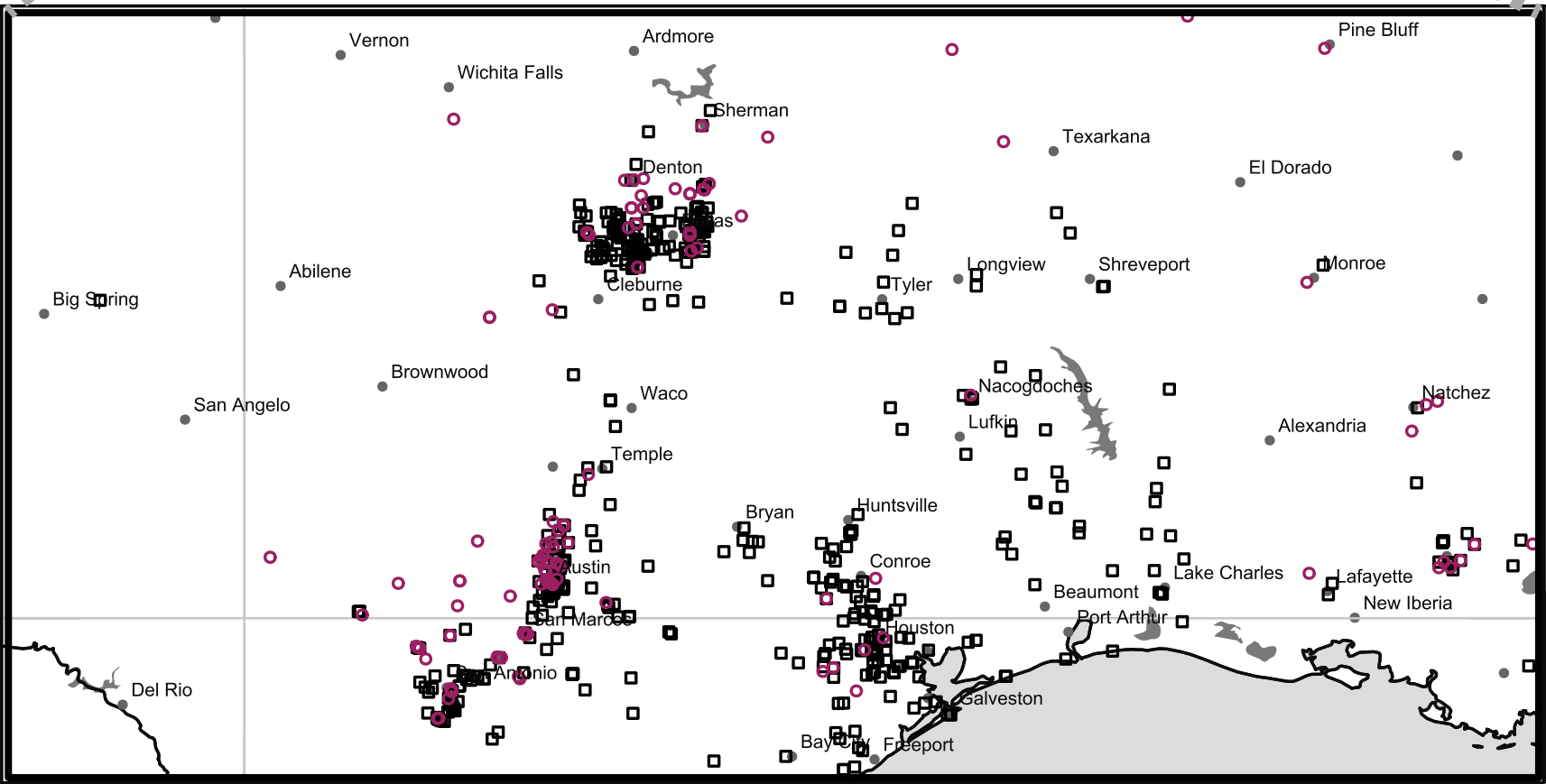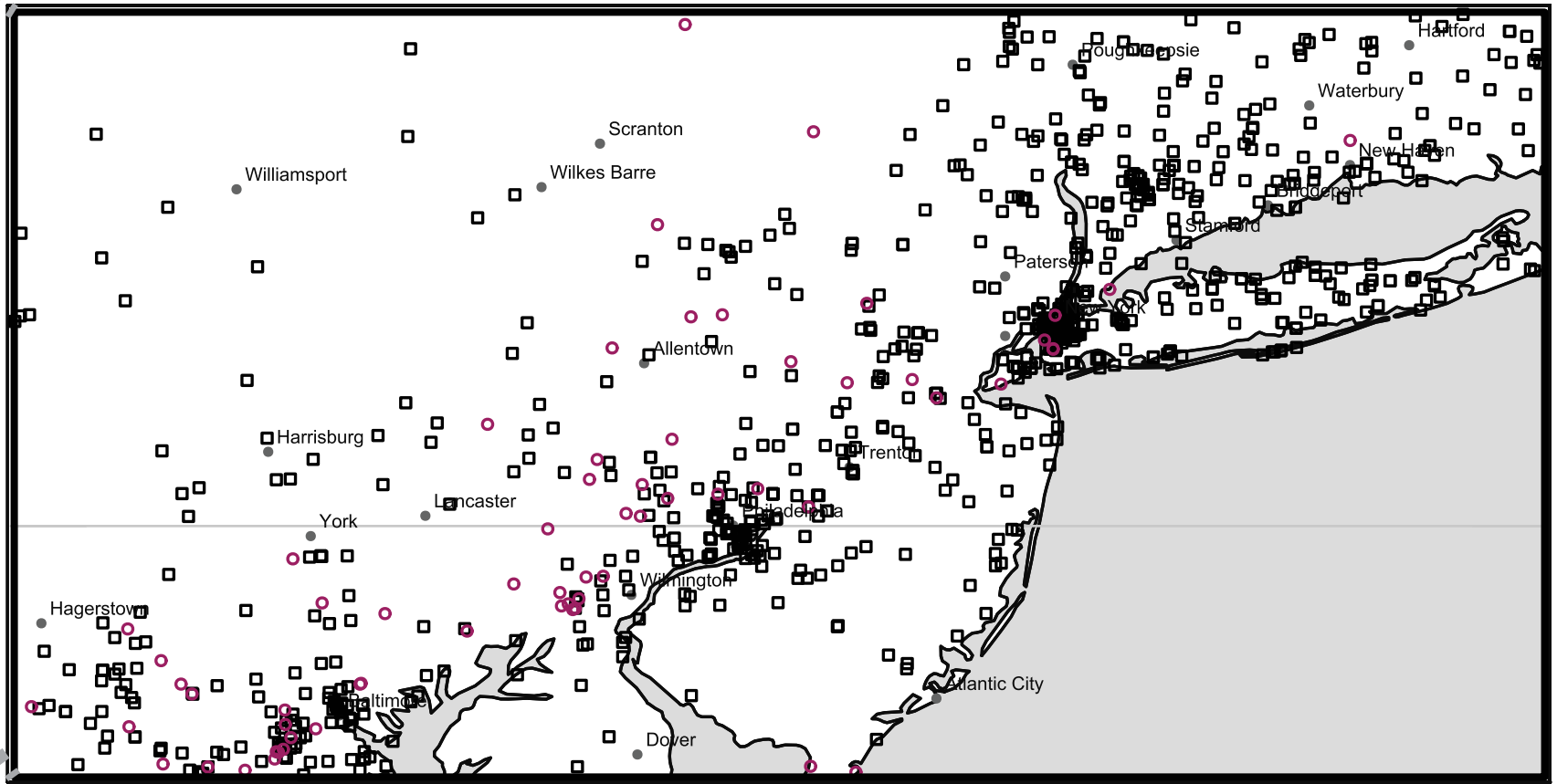

Supplement: S1 Fig — Red and black morph fall webworms occur sympatrically at a regional and local level, implicating a sympatric form of speciation. Regions shown are southern Ontario, northeastern USA, and southern Texas. All maps generated using Simplemappr (https://www.simplemappr.net/), for which maps are available in the public domain without license. (PDF) [file pone.0288415.s002.pdf]

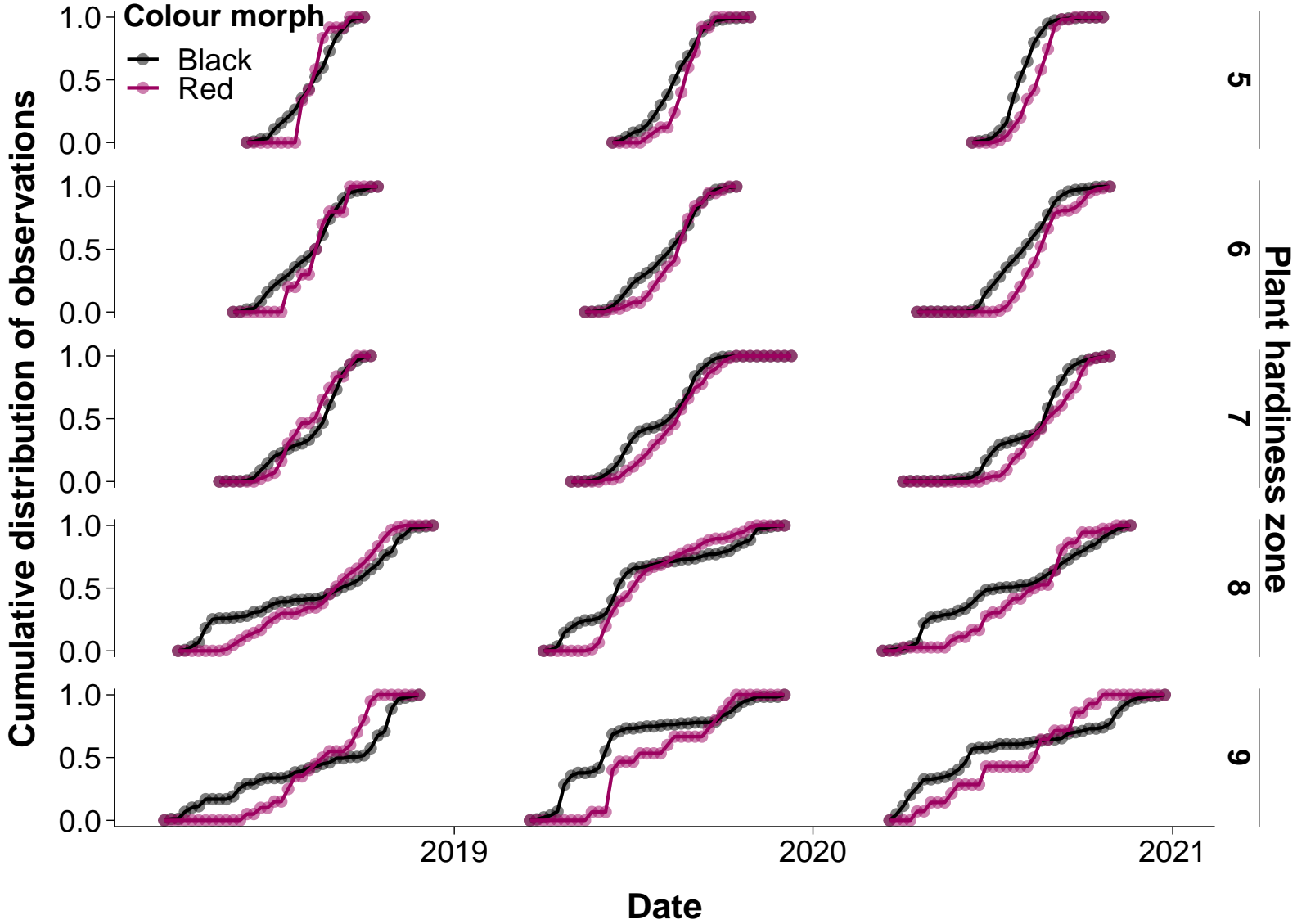

Supplement: S2 Fig — Points indicate cumulative proportion of observations, while lines show trends in distribution across years. (PDF) [file pone.0288415.s003.pdf]

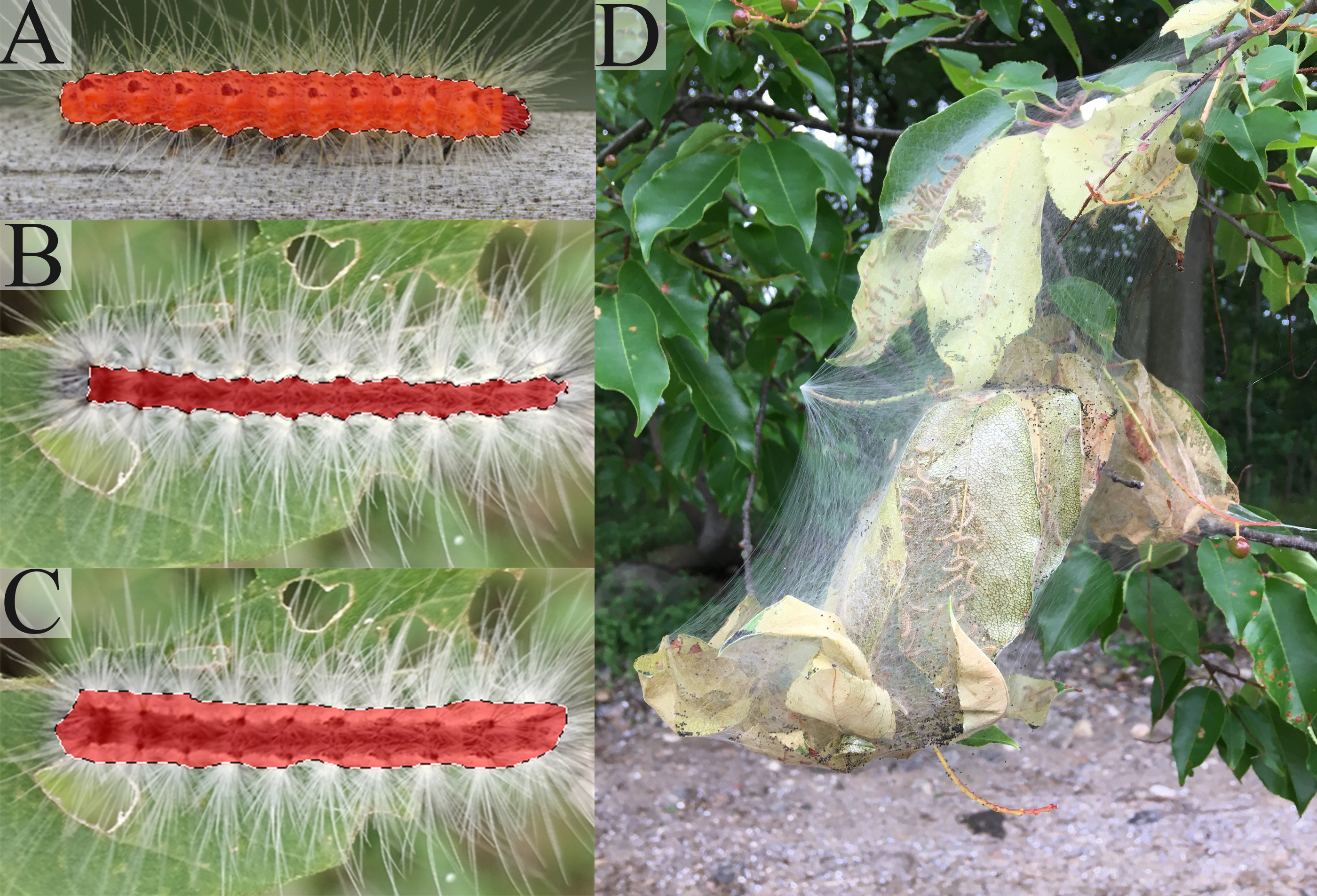

Supplement: S3 Fig — A) Complete isolation of fall webworm larva by Photoshop using object detection tool, no manual adjustment needed. B) Incomplete selection of fall webworm larva by object detection tool. Corrected manually by researcher using freehand lasso tool. C) Complete selection of fall webworm larva in Photoshop after manual correction using freehand lasso tool. D) Example of photo removed from colour phenotype analysis. Photo was low resolution, had poor exposure, was taken from a far distance, and had an object obstructing the webworms. Photos shown here obtained from iNaturalist.org under public domain licensing (CC0). (PNG) [file pone.0288415.s004.png]

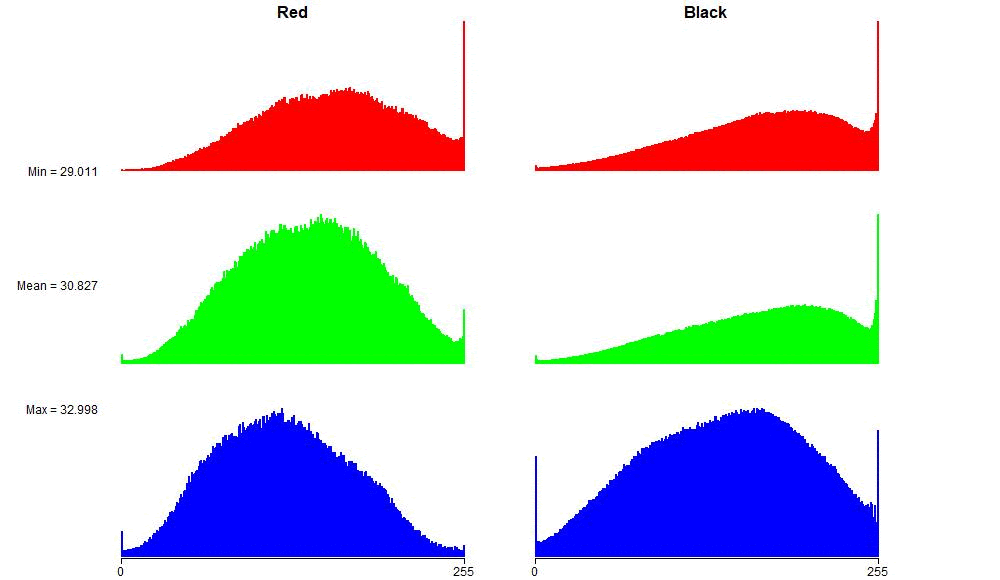

Supplement: S4 Fig — (GIF) [file pone.0288415.s005.gif]
